# Pharmaceutical cost management in an ambulatory setting using a risk adjustment tool

**DOI:** 10.1186/1472-6963-14-462

**Published:** 2014-10-21

**Authors:** David Vivas-Consuelo, Ruth Usó-Talamantes, Natividad Guadalajara-Olmeda, José-Luis Trillo-Mata, Carla Sancho-Mestre, Laia Buigues-Pastor

**Affiliations:** Research Centre for Health Economics and Management, Universitat Politècnica de València, Edificio 7 J, Campus de Vera s/n, 46022 Valencia, Spain; Valencian Health Department (Conselleria de Sanitat), General Directorate of Pharmacy and Pharmaceutical Products, Valencia, Spain

**Keywords:** Risk adjustment, Predictive models, Pharmaceutical expenditure, Chronic condition, Clinical Risk Groups, Capitation payments

## Abstract

**Background:**

Pharmaceutical expenditure is undergoing very high growth, and accounts for 30% of overall healthcare expenditure in Spain. In this paper we present a prediction model for primary health care pharmaceutical expenditure based on Clinical Risk Groups (CRG), a system that classifies individuals into mutually exclusive categories and assigns each person to a severity level if s/he has a chronic health condition. This model may be used to draw up budgets and control health spending.

**Methods:**

Descriptive study, cross-sectional. The study used a database of 4,700,000 population, with the following information: age, gender, assigned CRG group, chronic conditions and pharmaceutical expenditure. The predictive model for pharmaceutical expenditure was developed using CRG with 9 core groups and estimated by means of ordinary least squares (OLS). The weights obtained in the regression model were used to establish a case mix system to assign a prospective budget to health districts.

**Results:**

The risk adjustment tool proved to have an acceptable level of prediction (R^2^ ≥ 0.55) to explain pharmaceutical expenditure. Significant differences were observed between the predictive budget using the model developed and real spending in some health districts. For evaluation of pharmaceutical spending of pediatricians, other models have to be established.

**Conclusion:**

The model is a valid tool to implement rational measures of cost containment in pharmaceutical expenditure, though it requires specific weights to adjust and forecast budgets.

## Background

The growth in pharmaceutical cost is a real problem in health care sustainability [1]. This is due to a number of factors: ageing of population, introduction of new medicines and changes in prescription practices and age-related patient complexity. Furthermore, studies focusing on a clear understanding of pharmaceutical consumption, cost and morbidity patterns are needed to implement effective cost control.

One of the widest used tools for cost control in health expenditure is that of risk adjustment, used to make capitation finance systems. These can be found worldwide for both clinical and pharmaceutical management. In health systems where competition between insurance companies exists, such as USA and Germany, capitation attempts to avoid adverse risk selection. In other countries with comprehensive national health systems, such as UK and Sweden, capitation is used for an equitable distribution of resources [2].

Early approaches to adjustment of health expenditure were based on demographic variables alone. However, the introduction of other clinical variables related with population health statuses has improved this adjustment in several countries.

The work of Mossey and Roos [3] was the starting point for different studies that use disease related cost for risk adjustment, using information from insurance companies. The first diagnostic based models for forecasting health expenditure were introduced in the 80s, employing AAPC (Adjusted Average Per Capita Cost) [4] and DCG (Diagnostic Cost Groups) [5].

In the last 20 years, various studies have been carried out on the use of cost indicators based on information available from electronic records previously used by clinical services. The three best known Diagnostic Based Risk Adjustment Systems (DBRAS) are: Diagnostic Cost Groups/Hierarchical Coexisting Conditions (DCG/HCC) developed by Pope *et al*. [6, 7], Adjusted Clinical Groups (ACG) developed by Starfield *et al*. [8] and Weiner *et al*. [9] at Johns Hopkins University in Baltimore and Clinical Risk Groups (CRG) developed by Hughes *et al*. [10]. All of these are based on the International Classification of Disease, 9th Revision, Clinical Modification (ICD-9-CM), the codes of which are recorded electronically. While the DCG is based on cost, ACG and CRG were developed to measure health statuses.

According to a study by Berlinguet, Preyra and Dean [11], the CRG have greater clinical relevance while offering a predictive power similar to the other two systems (ACG and DCG/HCC).

Another important classification system is the Chronic Disease Score (CDS), developed by Von Korff *et al*. [12] using pharmaceutical consumption to identify chronic conditions of patients. This uses pharmacy databases to estimate disease prevalence in the absence of diagnostic information. Moreover, these databases have the advantage of generally being complete, precise and reliable, while codification of diagnostics may only register those conditions treated during a clinical visit or hospital stay and, as such, not reflect other important chronic conditions [13].

Various later studies have analysed the validity of the four health state indicators above, perfecting them and adapting them to each specific situation. Thus, different models of pharmaceutical expenditure were obtained from the CDS [14–19], DCG/HCC system [20, 21].

Although the ACG [22, 23], and CRG systems were developed to measure health status, their validity in explaining pharmaceutical expenditure has also been demonstrated [24–26].

In the United States, Medicare uses the developed DCG/HCC model. In 2006 they implemented the model, CMS (Centres for Medicare and the Medicaid) prescription drug hierarchical condition categories RxHCC [27]. For capitation payments the CMS-HCC model [7], based only on diagnostics, is used. The use of differentiated models for capitation and medicine payments is based on the findings of Zhao *et al*. [28], who give better predictive power for future prescription drug costs for mixed models that combine diagnostic and drug use data.

Other countries have their own system, such as that in Germany, where a morbidity based risk adjustment was introduced in 2009, embedded in a broader reform of the statutory health insurance system. The new formula covers 80 “severe” or “costly and chronic” diseases structured in a system of hierarchical groups [29].

The CRG system in Spain was first implemented in the Baix Empordà Health Service (*Serveis de Salut del BaixEmpordà*) [30], and projects are under way in the Autonomous Communities of Catalonia and Madrid. Other Autonomous Communities like the Pais Vasco [31] and some health centres in the Balearics [32] have opted for ACG. Regardless of the system used, all these suppose a significant advance on previous systems based on epidemiological variables [33] or prescriptions [14].

Over recent years, the Valencian Community (VC) (East coast of Spain) has been interested in adapting health expenditure by linking it with population morbidity. Firstly, the General Directorate of Pharmaceutical and Health Products (DGFPS) of the Valencian Health Department designed a standardised amount indicator [34] which offered primary health care pharmaceutical expenditure data per patient covered in a year. This allowed the standardisation of the population based on two categories: patients covered by the health system who have the right to free medication (fundamentally pensioners) and patients covered by the system who must pay part of the cost of the medication from the pharmacy. According to this, in 2011 primary health care pharmaceutical expenditure per standardised patient was 338 Euros, compared with the 275 Euros obtained without standardisation.

This indicator supposes an improvement regarding precision of results and was achieved thanks to the development of appropriate information systems and more specifically that of the primary health care electronic health record [35].

Vivas *et al*. [36] developed a model using Anatomical Therapeutic Chemical Classification (ATC) from electronic prescriptions drug data to explain pharmaceutical expenditure at a local level.

Since 2010, the DGFPS has been developing a system of patient classification in the VC based on CRGs, which allows stratification of patients according to morbidity. The differences between the use of data from prescriptions and diagnostics that have been observed in the DCG models do not affect the models based on CRG. The predictive power of CRG for future pharmaceutical expenditure has been demonstrated in three prior studies [24–26].

The goal of this work is to obtain a concurrent model of primary health care pharmaceutical expenditure for the entire region using CRGs. From this model we obtain weights for primary health care pharmaceutical expenditure per inhabitant and year based on the CRG, which may then be used to draw up budgets for the following year and control health spending in the 24 health districts of VC.

## Methods

### Data

The data was taken from the Population Information System’s (PIS) database of all patients registered and assigned to one of the 24 health districts in the VC, a region of 4.7 million inhabitants for the period 1^st^ Jan to 31^st^ Dec for 2012 and 2013. Although the initial number of patients was 5.2 million, 400,000 were discarded as non-residents with a stay of less than one month, leaving a total of 4.7 million for analysis. All information was made anonymous according to data protection regulations and our study was approved by the Behavioural Research Ethics Board at the *Generalitat Valenciana*.

For each patient we obtained the following information. Socio-demographic data: patient anonymisation code, age, sex, health centre, area, health district, PIS state (active/inactive) and pharmacy status (with or without co-payment). Usage data needed for the CRG grouper were: number of contacts in primary health care, number of hospital admissions, and days in hospital per admission (main diagnosis, coded according to ICD-9-CM). CRG data: CRG Base, ACRG1, ACRG2, ACRG3 (Table 1). Pharmaceutical cost data: cost of medicines according to the invoicing nomenclature of the Ministry for Health, Social Policies and Equality. These costs refer to the pharmaceutical expenditure in primary health care centres, which means the total cost is not included for health statuses 8 and 9, given that the majority of these medicines are provided by the hospitals.

**Table 1:** **Population** (**N**), **annual pharmaceutical expenditure in Euros and age by CRG core health status and severity level** (**ACRG3**) **2012**

ACGR3		N	%N	Average expenditure	Total expenditure	Average age	St. Dv. age
**1. Healthy**							
10	Healthy	1,633,686	35.10%	42.63	69,644,034	28.56	19.05
11	Healthy Non-User	784,168	16.85%	0.00	0	36.21	16.99
12	Delivery without Other Significant Illness	28,921	0.62%	49.30	1,425,805	30.56	8.41
14	Pregnancy without Other Significant Illness	19,451	0.42%	35.92	698,680	31.26	6.93
15	Evidence of Significant Chronic or Acute Diagnosis without Other Significant Illness	152,488	3.28%	190.76	29,088,611	40.35	23.00
**2. History of significant acute disease**							
20	History Of Significant Acute Disease	193,19	4.15%	95.96	1,853,851	33.23	19.65
22	Delivery with History of Significant Acute Illness	15,875	0.34%	209.51	3,325,971	33.87	10.84
24	Pregnancy with History of Significant Acute Illness	7,108	0.15%	64.70	459,888	31.83	5.99
25	Evidence of Significant Chronic or Acute Diagnosis with History of Significant Acute Illness	44,836	0.96%	166.72	7,475,058	38.06	21.98
**3. Single minor chronic disease**							
31	Single Minor Chronic Disease Level - 1	403,148	8.66%	190.25	76,698,907	45.58	18.58
32	Single Minor Chronic Disease Level - 2	41,173	0.88%	258.48	10,642,397	43.19	19.23
**4. Minor chronic disease in multiple Organ Systems**							
41	Minor Chronic Disease In Multiple Organ Systems Level - 1	90,903	1.95%	339.85	30,893,385	55.72	15.69
42	Minor Chronic Disease In Multiple Organ Systems Level - 2	25,3	0.54%	515.28	130,366	61.58	13.52
43	Minor Chronic Disease In Multiple Organ Systems Level - 3	34,801	0.75%	520.02	18,097,216	57.91	15.38
44	Minor Chronic Disease In Multiple Organ Systems Level - 4	5,204	0.11%	704.89	3,668,248	59.07	14.99
**5. Single dominant or moderate chronic disease**							
51	Single Dominant Or Moderate Chronic Disease Level - 1	484,232	10.40%	528.68	256,003,774	54.76	21.09
52	Single Dominant Or Moderate Chronic Disease Level - 2	135,731	2.92%	739.94	100,432,796	54.37	22.66
53	Single Dominant Or Moderate Chronic Disease Level - 3	39,795	0.86%	1108.05	44,094,850	59.30	19.73
54	Single Dominant Or Moderate Chronic Disease Level - 4	5,133	0.11%	1061.65	5,449,449	63.83	21.39
55	Single Dominant Or Moderate Chronic Disease Level - 5	9,556	0.21%	1348.04	12,881,870	66.70	17.46
56	Single Dominant Or Moderate Chronic Disease Level - 6	761	0.02%	1961.34	1,492,580	51.56	18.79
**6. Chronic disease in 2 or more Organ Systems**							
61	Significant Chronic Disease In Multiple Organ Systems Level - 1	217,363	4.67%	994.26	216,115,336	66.93	15.42
62	Significant Chronic Disease In Multiple Organ Systems Level - 2	93,125	2.00%	1362.09	126,844,631	69.14	14.57
63	Significant Chronic Disease In Multiple Organ Systems Level - 3	62,212	1.34%	1567.73	97,531,619	70.90	13.95
64	Significant Chronic Disease In Multiple Organ Systems Level - 4	40,647	0.87%	1835.01	74,587,651	72.87	13.26
65	Significant Chronic Disease In Multiple Organ Systems Level - 5	19,282	0.41%	2074.03	39,991,446	75.06	13.02
66	Significant Chronic Disease In Multiple Organ Systems Level - 6	2,259	0.05%	2087.73	4,716,182	75.24	13.40
**7. Dominant Chronic Disease in 3 or more Organ Systems**							
71	Dominant Chronic Disease In Three Or More Organ Systems Level - 1	7,202	0.15%	1861.56	13,406,955	73.70	10.35
72	Dominant Chronic Disease In Three Or More Organ Systems Level - 2	5,913	0.13%	2159.61	12,769,774	74.88	10.54
73	Dominant Chronic Disease In Three Or More Organ Systems Level - 3	12,577	0.27%	2420.64	30,444,389	76.04	10.33
74	Dominant Chronic Disease In Three Or More Organ Systems Level - 4	3,587	0.08%	2677.64	9,604,695	77.33	9.95
75	Dominant Chronic Disease In Three Or More Organ Systems Level - 5	2,694	0.06%	2784.93	7,502,601	77.07	10.08
76	Dominant Chronic Disease In Three Or More Organ Systems Level - 6	1,076	0.02%	2528.73	2,720,913	76.05	10.06
**8. Dominant and Metastatic Malignancies**							
81	Dominant, Metastatic, And Complicated Malignancies Level - 1	1,677	0.04%	800.04	1,341,667	61.41	16.28
82	Dominant, Metastatic, And Complicated Malignancies Level - 2	5,692	0.12%	1223.61	6,964,788	62.59	16.67
83	Dominant, Metastatic, And Complicated Malignancies Level - 3	6,419	0.14%	1649.05	10,585,252	66.99	14.72
84	Dominant, Metastatic, And Complicated Malignancies Level - 4	4,878	0.10%	2021.68	9,861,755	69.61	13.66
85	Dominant, Metastatic, And Complicated Malignancies Level - 5	1,389	0.03%	2131.74	2,960,987	70.86	12.19
**9. Catastrophic conditions**							
91	Catastrophic Conditions Level - 1	1,895	0.04%	1632.93	3,094,402	44.83	22.15
92	Catastrophic Conditions Level - 2	3,647	0.08%	1164.40	4,246,567	46.30	16.97
93	Catastrophic Conditions Level - 3	1,857	0.04%	1944.29	3,610,547	53.82	19.86
94	Catastrophic Conditions Level - 4	2,02	0.04%	1949.96	393,892	52.68	16.77
95	Catastrophic Conditions Level - 5	976	0.02%	2244.09	2,190,232	63.49	16.67
96	Catastrophic Conditions Level - 6	515	0.01%	2368.46	1,219,757	65.04	16.37
**Total**		**4,** **654,** **362**	**100%**	**298.71**	**1,** **301,** **898,** **793**	**41.** **32**	**23.07**

### Data sources

Data for the study was obtained from the electronic health record for primary health care (SIA) and the Minimum Data Set (MDS) of hospitals. Data for primary health care pharmaceutical expenditure was obtained from the prescription module of the Pharmaceutical Provision Manager, GAIA. The full amount of each prescription expended during the study period was accounted for.

### CRG calculation

To obtain the CRG we used 3 M™ Clinical Risk Grouping Software v.1.4. CRGs capture the resource utilization of all inpatient and ambulatory encounters. The groups identify individuals with multiple chronic co-morbid conditions and explicitly specify the severity of illness for each individual. The CRG system maps each diagnosis to one of 1,079 CRG groups that are similar in terms of relative severity, persistence, or recurrence, and health care resource expectations. CRGs, at the discretion of the user, can then be aggregated in order to reduce the number of groups. There are three tiers of aggregation. These are identified as ACRG1, ACRG2, and ACRG3 (Table 1). Each one progressively reduces the number of groups while maintaining, albeit with some adjustment, severity leveling. In the designed models we use 8 dummy variables - one for each core health status - plus 6 dummy variables for severity levels.

### Statistical analysis

With the data for 2012, concurrent regression models were made using the total pharmaceutical expenditure by patient and year (C) as dependent variable. As C was not normally distributed, C is ln-transformed as a better approach to its normal distribution [37].

As 420,000 patients (8%) had cost 0, which results in ln -inf, the final dependent variable was considered to be C + 1, resulting in all cost values < 1 having a positive Ln value. Prediction from these models must then be retransformed by subtracting -1, to obtain estimates on the original scale.

Six models were made using the 2012 data, combining the following independent variables in each model:

(i) age and sex (1 male and 0 female);
(ii) age, sex, and 8 CRG core health statuses;
(iii) 8 CRG core health statuses alone;
(iv) 8 CRG core health statuses, only for the paediatric cohort;
(v) 8 CRG core health statuses excluding paediatrics cohort;
(vi) 8 CRG core health statuses, 6 severity levels, age and sex, excluding paediatric population.

In all models except (i), the healthy group (Health Status 1) was the control variable necessary in the regression model. Models (i), (ii) and (vi) were designed to observe if there is any difference between age and sex groups.

The models were estimated by means of ordinary least squares (OLS). The goodness of fit was determined through the corrected R^2^ value and the F Snedecor, mean squared error, and for each of β coefficients t-Student values were obtained to determine the level of significance. The assumptions of normality, homoscedasticity and linearity were considered. Split analysis validation to avoid overfitting was carried out. We used a random sample of 70% of the study subjects for model development and 30% for model validation.

The weight of each health status according to the pharmaceutical expenditure for this status was obtained by retransforming the predicted ln expenditures back into nominal using the smearing estimate provided by Duan (1983) [38]. With this nonparametric retransformation the homoscedastic error is corrected. Thus, the expression for this smearing estimate is:
1

Where:

ϵ= the residual errors.

n = number of observations.

With model iii selected to predict the cost, we established a case mix (CM) system based on the weights of each of the health statuses. Thus, it is possible to calculate the CM for the region and each health district and compare the allocated budget with real expenditure.

The equation for *CM*_*j*_ calculation for each health district j is:
2

Where,

*N*_*ij*_ = Number of population of group *i* in the health district *j*.

*W*_*i*_ = Weight of each *i* core health status group.

The process used for obtaining a predictive budget by health district was the following: Firstly, the weights were calculated for 2012 using the regression model with real data to establish the relative consumption for each CRG. Through the expression (2) the CM is calculated. The Health Authority then established an overall budget for pharmaceutical expenditure for 2013, partly by taking into account the prior year’s expenditure. Applying the weights, we calculated the number of adjusted patients, and divided the overall budget by this number, obtaining the standard price of an adjusted patient. This allows us to give a budget to each doctor or district according to the number of adjusted patients they have.

The model weights are recalibrated annually to introduce possible changes relating to health status, price changes and clinical practice. A cap is established, however, for maximum target budget. That is, a cost per patient adjusted by morbidity is set each year which is used to establish the budget.

## Results

### Patient stratification and pharmaceutical expenditure

**Figure 1:**
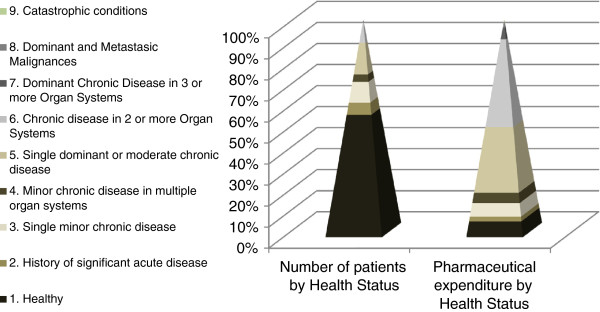
**Stratification of patients by core health status and pharmaceutical expenditure in 2012.** Shows a graph for the number of patients, classified into each of the nine CRG core health statuses, and the average cost in this period. In the graph for this data it can be clearly seen how each strata of the population is related to the pharmaceutical expenditure.

Table 1 shows the number of patients, classified into each of the nine CRG core health statuses, percentage of patients and the average cost for the year 2012. In the graph for this data (Figure 1) it can be clearly seen how each strata of the population is related to the pharmaceutical expenditure. Health Status 6 (chronic disease in 2 or more organ systems) represents 48% of total primary health care pharmaceutical expenditure despite only representing 11% of population.

Health status 5 (single dominant or moderate chronic disease) represents 26.4% of total primary health care pharmaceutical expenditure while only representing 15.1% of population. These two statuses together account 74.4% of total expenditure (Figure 1).

### Regression models

In Table 2 we show the model coefficients and other statistics. Of the proposed models, model (vi) achieved the best fit, with an R^2^ of 60.3%.

**Table 2:** **Results of different predictive models for pharmaceutical expenditure per year and patient** (**C**) **in Euros 2012**

	Model i age & sex	Model ii CRG core health status, age & sex	Model iii CRG core health status	Model iv CRG core health status	Model v CRG core health status	Model vi CRG core health status, severity level, age & sex
Variables	Total population	Total population	Total population	Under or equal 14	14 PLUS	14 PLUS
Constant	1.159	1.391	1.736	1.865	1.697	.906
Age	.057	.016				.026
Sex	-.461	-.324				-.388
Health status 2		1.751	1.816	1.262	1.955	1.873
Health status 3		2.179	2.440	1.548	2.537	2.214
Health status 4		3.201	3.691	2.134	3.743	2.951
Health status 5		3.399	3.799	2.119	3.954	3.291
Health status 6		4.352	4.992	3.120	5.048	3.946
Health status 7		5.055	5.760	3.920	5.801	4.400
Health status 8		4.325	4.870	1.891	4.946	3.713
Health status 9		4.217	4.493	3.129	4.608	3.785
Severity level 2						.406
Severity level 3						.613
Severity level 4						.701
Severity level 5						.823
Severity level 6						.878
F	883600.05	613728.44	711221.29	17380.11	650280.64	396142.59
N	4,654,362	4,654,362	4,654,362	739,525	3,914,837	3,914,837
R^2^	**0.275**	**0.569**	**0.550**	**0.158**	**0.571**	**0.603**

Furthermore, within the Spanish health system it is important to analyse the pharmaceutical expenditure for patients under 14, as these patients are attended by paediatricians in primary health care. Applying the CRG model (iv) to this cohort, we found a very low level of explanation, 15.8%. Therefore a special predictive model must be developed for these patients.

In spite of models (ii), (v) and (vi) being better, we have taken the coefficients from model (iii), the R^2^ of which is 55% for reasons of operational and practical use and understanding by clinical users.

For the CM system implemented, relative weights were established by retransforming the coefficient for each health state through the smearing estimator and adding the value of 1 as presented in Table 3. The result of the smearing estimator (γ), the mean of the anti-ln of the residuals, was 1.693 (expression 1).

**Table 3:** **Calculation for weights by CRG core health status from model 2012**

Core health status	Ln (C + 1) (a)	Expenditure C (b)	Standard weight (c)
1. Healthy	1.736	8.607	1
2. History of significant acute disease	3.552	58.068	6.75
3. Single minor chronic disease	4.176	109.242	12.69
4. Minor chronic disease in multiple organ systems	5.427	384.212	44.64
5. Single dominant or moderate chronic disease	5.535	427.851	49.71
6. Chronic disease in 2 or more Organ Systems	6.728	1413.267	164.21
7. Dominant Chronic Disease in 3 or more Organ Systems	7.496	3048.575	354.21
8. Dominant and Metastasic Malignances	6.606	1250.432	145.29
9. Catastrophic conditions	6.229	858.032	99.69

(a) = Ln (C + 1).

(b) = (1.693 e^a^)-1.

(c) = b_i_/b_1_.

It should be noted that for groups 8 and 9 we obtained lower weights than given by the original CRG. This is due to patients in these groups principally using hospital dispensaries, while our study drew data from primary health care only. These patients suffer from malignancies and catastrophic diseases such as renal failure or organ transplants.

### Analysis by health district

**Figure 2:**
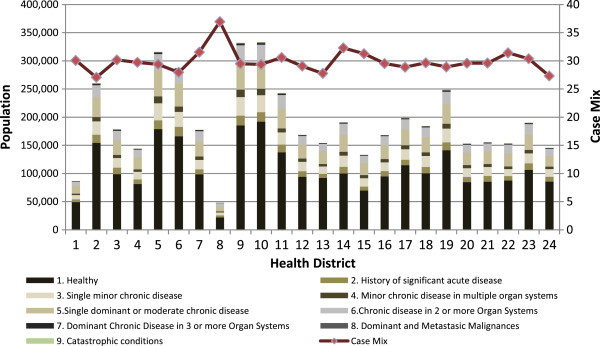
**CRG core health status by health district and case mix 2012.** Shows a stacked column chart, comparing the contribution of each value to a total across categories of CRGs core health statuses for each health districts. The x axis of the chart shows the health districts compared and the y axis represent a double scale with the case mix on the right and the n° of patient grouped by CRG core health status on the left.

Figure 2 shows the number of patients assigned to each health district, grouped in health statuses. The line represents the CM in the health departments, calculated as the summation of equivalent patients divided by real patients in each health district, expression (2).

**Figure 3:**
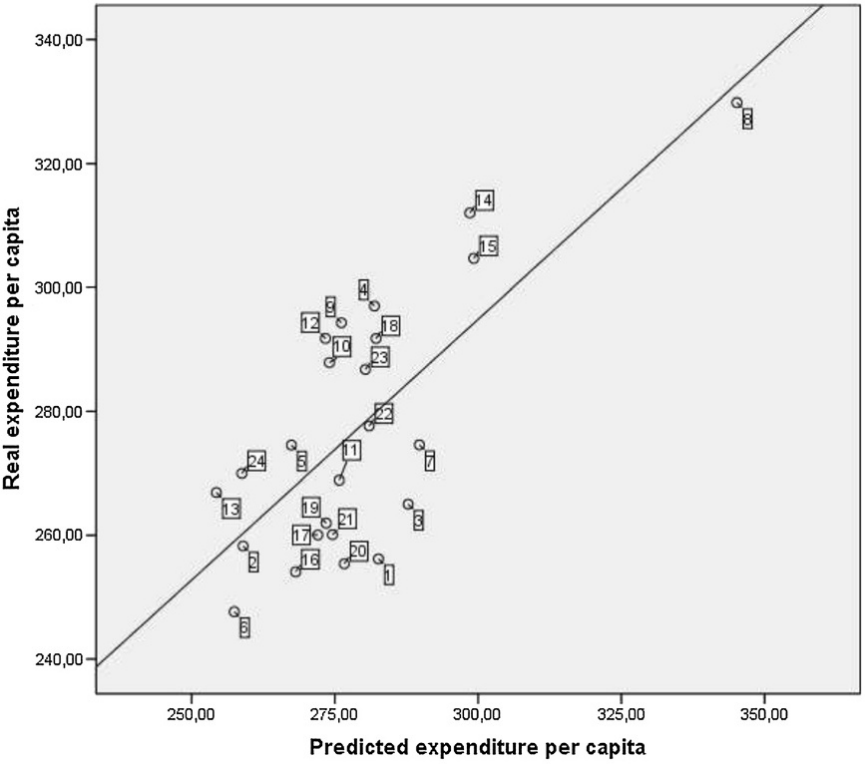
**Pharmaceutical expenditure real and predicted by health district 2013.** Shows a scatter plot to display values for real (y axis) and predicted (x axis) pharmaceutical expenditure of each heath district.

Figure 3 shows the relation between predicted expenditure according to CM and real pharmaceutical expenditure in primary care for each health district. 13 health districts have spent less than was estimated by the proposed model considering the health status of patients assigned to them, and 11 have incurred higher costs than predicted. This means that with the same equivalent patients, some departments generate higher outpatient pharmaceutical spending than others and some health departments are managing pharmaceutical spending better than others (Table 4).

**Table 4:** **Real and predicted pharmaceutical expenditure and case mix adjusted by health district in 2013**

Health district	Case mix (1)	Real C (2)	Predicted C (3)	Population. N (4)	Average real C (5) = (2)/(4)	Average predicted C (6) = (3)/(4)	Differences (7) = (5)-(6)
1	35.29	22,404,536.70	24,714,441.39	87,448	256.20	282.62	-26.41
2	32.34	68,608,549.95	68,793,348.13	265,636	258.28	258.98	-0.70
3	35.94	47,720,043.15	51,816,313.29	180,051	265.04	287.79	-22.75
4	35.20	43,430,451.48	41,221,848.93	146,219	297.02	281.92	15.10
5	33.39	88,177,744.42	85,893,722.31	321,192	274.53	267.42	7.11
6	32.15	72,489,772.23	75,357,459.28	292,715	247.65	257.44	-9.80
7	36.18	49,825,584.93	52,580,973.57	181,468	274.57	289.75	-15.18
8	43.10	15,989,816.02	16,733,570.96	48,479	329.83	345.17	-15.34
9	34.48	99,701,853.24	93,549,589.70	338,770	294.31	276.14	18.16
10	34.22	97,918,781.79	93,218,916.90	340,151	287.87	274.05	13.82
11	34.43	66,559,137.30	68,259,770.67	247,529	268.89	275.76	-6.87
12	34.13	49,736,535.24	46,595,511.68	170,456	291.79	273.36	18.43
13	31.76	41,575,756.05	39,612,929.41	155,755	266.93	254.33	12.60
14	37.28	60,142,256.18	57,547,721.39	192,759	312.01	298.55	13.46
15	37.36	40,973,176.21	40,243,795.62	134,490	304.66	299.23	5.42
16	33.48	43,043,816.92	45,423,640.85	169,389	254.11	268.16	-14.05
17	33.97	52,476,085.67	54,889,413.61	201,780	260.07	272.03	-11.96
18	35.24	54,265,489.06	52,482,585.31	185,984	291.78	282.19	9.59
19	34.15	66,260,672.07	69,172,538.68	252,932	261.97	273.48	-11.51
20	34.54	39,467,859.22	42,750,213.31	154,533	255.40	276.64	-21.24
21	34.29	41,080,531.21	43,366,934.60	157,932	260.12	274.59	-14.48
22	35.09	44,150,450.69	44,686,006.13	159,031	277.62	280.99	-3.37
23	35.01	55,746,290.13	54,498,692.53	194,401	286.76	280.34	6.42
24	32.31	40,153,602.94	38,488,854.55	148,732	269.97	258.78	11.19
Total	34.38	1,301,898,792.80	1,301,898,792.80	4,727,832	275.37	275.37	0.00

(1) District case mix (CM).

(2) Annual real cost.

(3) **Predictive expenditure**_health district_ = ***real expenditure***_***vc***_ ***CM***_***health district***_  ***N***_***health district***_/***CM***_***cv***_ ***N***_***vc***_.

(4) Health district total population.

## Discussion

The study results show the basis for a pharmaceutical management model designed to improve efficiency in the use of medicines and allocation of budgets. The main innovation is the linking of pharmaceutical expenditure to patient morbidity, a factor not introduced until now in the majority of European health systems.

Discussion points may be centred around three aspects: the reliability of the classification system, the predictive capacity of the developed model, and practical utility.

The reliability of CRGs with respect to correct patient stratification depends on the appropriate inclusion of the diagnostics in the Electronic Health Record (EHR). One of the indicators for evaluating this is the percentage of healthy patients, both users and non-users. The results presented here have greatly improved with respect to stratifications undertaken in the trial period and those presented by other authors. The deficiencies in the initial diagnostics code gave this group as being 60% of the population, whereas with correct coding the value is 52%, representing the real proportion (34% healthy users and 16% non-users). This indicates a substantial improvement in the codification. Other authors, using data from 2008, give this status to 70% of the population [30].

The proposed model uses population stratification into risk groups based on CRGs, but develops its own weights for CRG core health statuses. If we compare the predictive capacity of this model with others described in the bibliography that use other patient classification systems, we see that it reaches, at minimum, the same level of explanation in terms of R^2^ and t-student statistics. Thus, the models based on patient classification using ATC [36] achieve an R^2^ of 57%, the models based on ACG [23] 35.4%, and those that use DxRx-PMs [39] 42.6%.

As the goal of our model is to assign predictive budgets with an objective level of expenditure for the health districts and primary health care physicians, it should be noted that the general model does not serve for the population of under-14s attended by paediatricians. As such, this is one of the weaknesses of a CRG based model. This also occurs in the ACG system, as indicated by Aguado *et al.* [23], who note that in children there is greater variability among physicians and centres not related to case-mix.

For a target consumption based on forecasts by the model, there are two other variables to consider in pharmaceutical expenditure in budgetary adjustments: the cost of the medicines and the goals of the adjustment. Over recent years we have effectively seen drops of around 7% in the price of medicines, driven by the increase in the consumption of generics and the reduction in prices from the Ministry for Health, Social Policies and Equality. During the last three years there has been a decrease every month in the prices set by the Spanish Agency for Medicines (*Agencia Española de Medicamentos*).

The other factor is the adjustment of pharmaceutical expenditure made by the health authorities via the protocols of rational use that prevent multiple medication of a patient and the use of medicines not based on evidence, especially in chronic pathologies such as hypercholesterolemia or osteoporosis. As such, in establishing expenditure goals by district and doctor from the specific experience from the VC for 2013, the forecast expenditure of the model decreases by 15%.

We observed the need for advanced and efficient IT development as a condition for the introduction of this system. Firstly, for the stratification of patients using the CRG system and then a further programme to communicate the classifications and predictions to the health districts and health workers. The IT system developed allows us to know the chronic diseases and co-morbidity of the patients included in each health status. This is of the greatest use when managing patients in programmes for the most prevalent chronic diseases. The system is linked to the EHR, making it possible to know all the diagnoses, treatments, hospital stays, etc.

## Conclusions

The developed model based on CRGs can be of great use in managing the pharmaceutical spending in integrated health services such as the National Health Service (NHS). In future development hospital pharmaceutical expenditure must be included to better explain the weights of statuses 8 and 9.

The predictive power of the developed model is similar to other models based on diagnostics, validating its use in managing primary health care pharmaceutical expenditure.

The general case-mix model is not applicable for establishing expenditure goals for paediatrics, as the CRG classification system is not valid for isolated patients of under 14 years of age.

The predictive models must consider adjustments which include variations in the price of medicines and rationalisation measures in pharmaceutical expenditure, so as to produce incentive-based targets.

## Authors’ information

David Vivas-Consuelo (DVC) and Natividad Guadalajara-Olmeda (NGO) are PhD Professors in the Research Centre for Health Economics and Management at Universitat Politècnica de València (Spain) and Carla Sancho-Mestre is a PhD candidate of the same university. José Luis Trillo-Mata (JLTM) and Ruth Usó-Talamantes (RUT) are Director and Deputy Director of the General Direction of Pharmacy and Pharmaceutical Products in the Valencian Health Departament (Spain). Laia Buigues-Pastor (LBP) works as an economist at the Pharmacoeconomics office in the same institution.
