# Erratum to: Pharmaceutical cost management in an ambulatory setting using a risk adjustment tool

**DOI:** 10.1186/s12913-017-2186-3

**Published:** 2017-03-31

**Authors:** David Vivas-Consuelo, Ruth Usó-Talamantes, Natividad Guadalajara-Olmeda, José-Luis Trillo-Mata, Carla Sancho-Mestre, Laia Buigues-Pastor

**Affiliations:** 1 Research Centre for Health Economics and Management, Universitat Politècnica de València, Edificio 7J, Campus de Vera s/n, 46022 Valencia, Spain; 2 Valencian Health Department (Conselleria de Sanitat), General Directorate of Pharmacy and Pharmaceutical Products, Valencia, Spain

## Erratum

Following the publication of this article [1] the authors would like to correct their ‘Acknowledgments’ section to read: “This work was supported by “Instituto de Salud Carlos III - Ministerio de Economía y Competitividad” and the European Union (FEDER funds) — FIS PI12/00037”. The authors would like to thank members (Juan Bru and Inma Saurí) of the Pharmacoeconomics Office of the Valencian Health Department. The opinions expressed in this paper are those of the authors and do not necessary reflect those of the afore-named. Any errors are the authors’ responsibility. We would also like to thank John Wright for the English editing”.
